# Protocol for a randomized controlled trial of mindfulness-based stress reduction to improve attentional control in older adults (HealthyAgers trial)

**DOI:** 10.1186/s12877-022-03334-7

**Published:** 2022-08-13

**Authors:** Ruchika Shaurya Prakash, Stephanie Fountain-Zaragoza, Megan Fisher, Oyetunde Gbadeyan, Rebecca Andridge, Janice Kiecolt-Glaser, Heena R. Manglani, Elizabeth J. Duraney, Anita Shankar, Michael R. McKenna, James Teng, Madhura Phansikar, Rosie Canter

**Affiliations:** 1 Department of Psychology, The Ohio State University, 62 Psychology Building, 1835 Neil Avenue, Columbus, OH 43210 USA; 2 Center for Cognitive and Behavioral Brain Imaging, The Ohio State University, Columbus, OH USA; 3 Department of Health Sciences and Research, Medical University of South Carolina, Charleston, SC USA; 4 National Centre for Healthy Ageing, Peninsula Clinical School, Faculty of Medicine, Monash University, Melbourne, Australia; 5 Department of Biostatistics, The Ohio State University, Columbus, OH USA; 6 Institute of Behavioral Medicine Research, Wexner Medical Center, Columbus, OH USA

**Keywords:** Mindfulness, Healthy aging, Attentional control, Mind-wandering

## Abstract

**Background:**

Mindfulness meditation is a form of mind–body intervention that has increasing scientific support for its ability to reduce age-related declines in cognitive functioning, improve affective health, and strengthen the neural circuitry supporting improved cognitive and affective health. However, the majority of existent studies have been pilot investigations with small sample sizes, limited follow-up data, and a lack of attention to expectancy effects. Here, we present the study design of a Phase I/II, efficacy trial—HealthyAgers trial—that examines the benefits of a manualized mindfulness-based stress reduction program in improving attentional control and reducing mind-wandering in older adults.

**Methods:**

One hundred fifty older adults (ages 65–85 years) will be randomized into one of two groups: an eight-week mindfulness program or an eight-week, placebo-controlled, lifestyle education program. Behavioral and neuroimaging assessments are conducted before and after the training. Participants are then invited to booster sessions once every three months for a period of 12 months with post-intervention follow-up assessments conducted at 6-months and 12-months. The primary outcomes for the study are behavioral measures of attentional control and mind-wandering. Additional, secondary outcomes include network strength in an a priori defined neuromarker of attentional control, fluid and everyday cognition, emotion regulation strategy use, and markers of inflammation.

**Discussion:**

This study will establish the efficacy of a group-based, low-cost mind–body intervention for the inter-related facets of attentional control and mind-wandering in older adults. Strengths of this study include a well-designed, placebo-controlled comparison group, use of web/mobile application to track study adherence, and longitudinal follow-up.

**Trial registration:**

Clinicaltrials.gov (#NCT03626532). Registered August 4, 2018.

**Supplementary Information:**

The online version contains supplementary material available at 10.1186/s12877-022-03334-7.

## Background

Older adults are a rapidly growing sector of the U.S. population as adults over the age of 65 years are projected to almost double from 56 million in 2020 (17% of the population) to 95 million in 2060 (23% of the population) [1]. Maintaining cognitive faculties is an important public health goal as cognitive functioning is highly related to well-established markers of physical health, like systemic inflammation [2, 3], and plays a critical role in activities that help maintain independent living, like driving [4], and in emotion dysregulation [5–7]. Thus, cognitive functioning has direct implicatons for the well-being and satisfaction of older adults [8]. Age-related declines in cognitive functioning have been noted across the domains of processing speed, working memory, executive control of attention, cognitive control, and episodic memory [9–11], with declines in some domains of cognition beginning as early as the mid-30s [12]. Moreover, behavioral shifts in measures of cognitive functioning are also accompanied by concomitant shifts in the functional and structural integrity of the aging brain with older adults showing neural dedifferentiation—the reduced recruitment of specialized canonical networks and nodes during cognitive tasks with increasing age [13, 14]. These age-related declines in cognitive functioning, both behavioral and neural representations, are thought to be undergirded by the increase in task irrelevant observations with advancing age. Older adults demonstrate a failure to maintain attentional sets, to computationally prioritize task-relevant information, and to limit attentional overprocessing of task-irrelevant information, resulting in notable declines in higher-order control operations [15–17].

However, despite a more nuanced and in-depth understanding of the cognitive and neural changes associated with aging, the health field’s capacity to translate these findings into pragmatic, efficacious remediation methods remains limited. Cognitive training programs show modest improvements in overall cognition with limited benefits to tasks of everyday functioning [18, 19]. Although studies of strategy-specific cognitive training are able to identify factors that may mediate the impact of the intervention for changes in cognition, there have been limited far-reaching transfer effects [20, 21]. Comparatively, multi-modal interventions, combining across various training modalities [22, 23] show the most promising effects. However, in these studies, it has been difficult to isolate the mechanisms of action. Thus, the scientific premise of the current study is based on an urgent need to explore alternative training programs that provide theoretical promise for reducing age-related cognitive decline, particulary in attentional overprocessing of task-irrelevant information. This could then have broad transfer effects on emergent cognitive operations of cognitive control, emotion regulation, and for domains of cognitive functioning that directly impact everyday functioning, like driving.

Mindfulness training, traditionally offered as an eight-week program called the mindfulness-based stress reduction (MBSR) [24], has gained increased popularity for its potential to improve cognition [25, 26], ameliorate symptoms of depression and anxiety [27], alter perceived and objective markers of stress [28], enhance emotion regulation [29], and alter the neural circuitry supporting cognitive and emotional functioning [30, 31]. Mindfulness, as defined in the contemporary sciences, is the practice of purposefully directing attention, in a non-judgmental way, to observe the unfolding of each moment as it takes place [32]. As such, the various components of attention, including the fluctuations in attention—quantified via the propensity to engage in mind-wandering—are being investigated for their malleability as a function of engagement in mindfulness practices. In our recent scoping review of the contemplative sciences literature examining the impact of mindfulness meditation for components of attention, we found the most robust evidence for mindfulness training to impact executive control of attention [33]. Moreover, results of a pilot, randomized controlled trial in our lab also provided evidence for mindfulness training to reduce off-task thinking, or mind-wandering quantified using both self-reported thought probes and response time variability in healthy older adults [34]. These reductions in mind-wandering propensity, replicated by other clinical trials (see Feruglio [35] for a systematic review), provide preliminary support that training in mindfulness meditation may strengthen the goal maintenance system, enhancing one’s ability to prioritize task-relevant representations, and to filter out irrelevant, internal thoughts or external stimuili in the environment.

Taken together, there is a growing recognition for the potential for mindfulness practices to improve attentional control—the biasing of task-relevant representations at the temporal, spatial, and response stages. However, this literature is still in its infancy, particularly in the evidence supporting mindfulness meditation as a tool for reducing age-related cognitive decline. The majority of studies, including our own pilot clinical trial, have had limited follow-up data, have relied on self-reported data for quantifying adherence to study materials, and did not include broad measures of transfer effects. This Phase I/II efficacy trial—HealthyAgers—aims to build on the existing literature by examining the immediate and longitudinal impact, relative to an active control group, of an eight-week mindfulness-based stress reduction (MBSR) program in improving attentional control and reducing mind-wandering in older adults. In addition, the control group in the study—Lifestyle Education (LifeEd) program—has been carefully designed to match the training group for non-specific factors that may influence the outcomes of interest. Participants in both groups will engage in a booster session every three months across the 12-month period following the intervention. Importantly, we have designed an in-house web/mobile application—HealthyAgers—to measure engagement with study materials for both groups to allow for careful assessments of dose–response relationships. We hypothesize that mindfulness training, compared to lifestyle education training, will improve attentional control and reduce mind-wandering. Secondary measures of this study will also assess the broad impact of mindfulness meditation for functional connectivity, fluid and everyday cognition, emotion regulation, and systemic inflammation. We hypothesize that mindfulness training will additionally result in an improvement in these secondary measures of healthy aging. The results of this study will establish the efficacy of this increasingly popular, low-cost intervention for older adults with regard to cognitive, affective, and neural health.

## Methods/design

### Study design

**Fig. 1:**
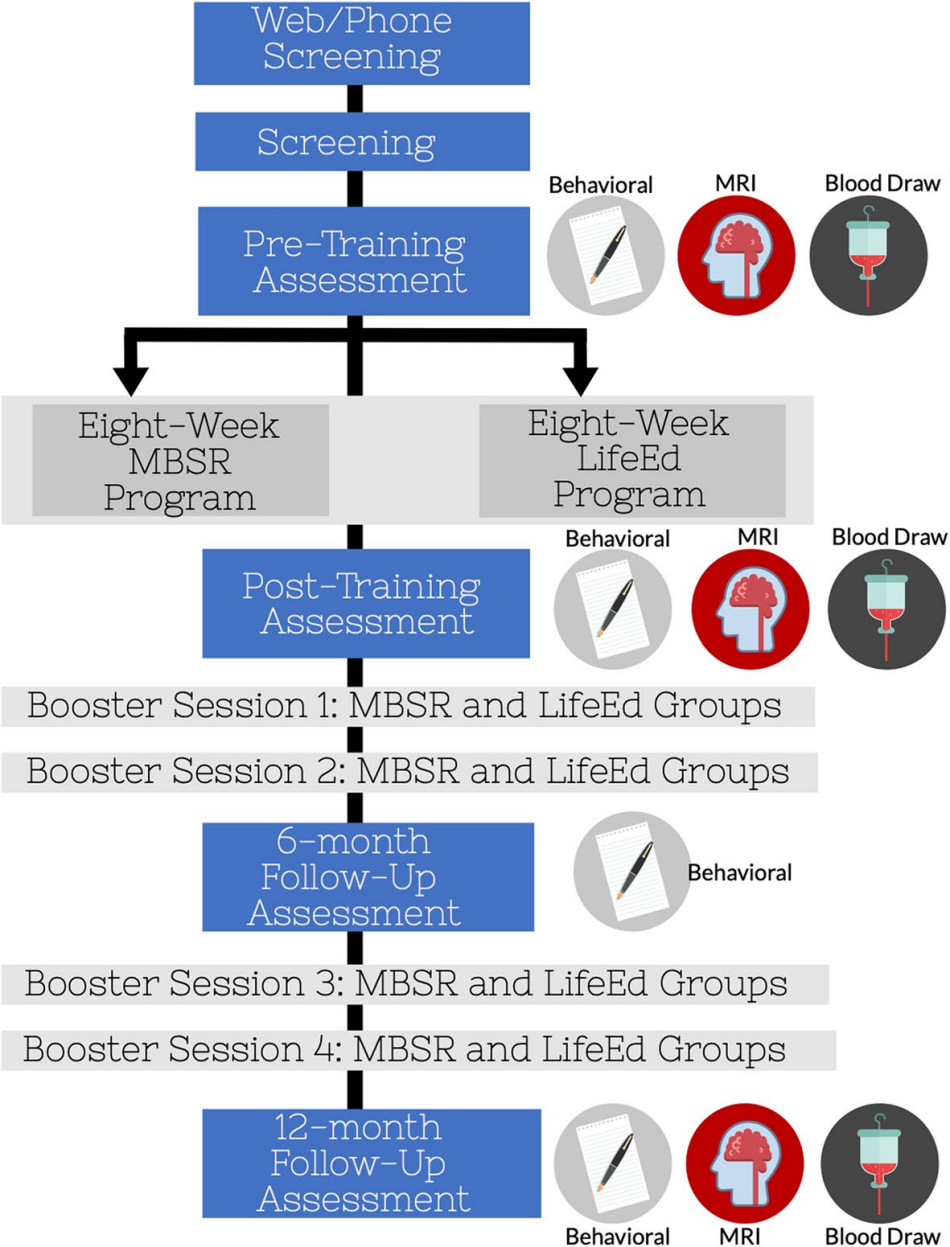
Flowchart of the HealthyAgers Trial

The proposed study is a single-blind, randomized controlled trial (RCT) designed to examine the differential impact of mindfulness training compared to a lifestyle education group (LifeEd) on behavioral and neural measures of attentional control and mind-wandering. Secondary assessments involve measures of fluid cognition, everyday cognition, emotion regulation, and systemic markers of inflammation. One hundred fifty older adults (65–85 years old) are being recruited for the study and randomized to either an eight-week MBSR group or an eight-week LifeEd control group. Behavioral, neuroimaging, and inflammation data will be conducted at baseline prior to randomization and again at the conclusion of the eight-week intervention. Further, participants will be followed for an additional 12 months, during which time booster sessions will be provided once every three months, with cognitive and affective assessments performed at 6-month and 12-month follow-ups. Neuroimaging and inflammation data will only be collected at the 12-month follow-up. Figure 1 illustrates a timeline of study participation.

### Setting

Assessments and the intervention will be conducted in the Psychology Building at The Ohio State University. The Department of Psychology houses individual neuropsychological testing rooms to allow for the convenient administration of neuropsychological and emotional regulation measures. All structural and functional MRI data will be collected at the Center for Cognitive and Behavioral Brain Imaging housed in the Department of Psychology at Ohio State. Prior to the onset of the COVID-19 pandemic, all intervention sessions were provided in the group therapy spaces housed in the Department of Psychology. These spaces were specifically designed for offering mindfulness and LifeEd sessions. Intervention sessions have been shifted to a virtual format following the COVID-19 pandemic, but assessment sessions continue to be collected in-person.

### Participant eligibility

Participants will only be included in the study if they satisfy the following criteria: aged between 65–85 years, corrected (far) visual acuity of 20/40 or better, have adequate hearing for assessment purposes, and have self-reported proficiency in spoken English and in reading comprehension. Additionally, we will only include participants if they are able to attend the majority of eight-week sessions and attend all assessment sessions, have a self-reported interest in engaging in mind–body interventions, can commit to a 30-min homework practice five times per week, and have the ability to engage in light stretching and toning exercises.

Additionally, participants will only be included in the study if they are classified as cognitively normal or having mild cognitive impairment (MCI) based on a battery of neuropsychological measures employed to screen individuals with dementia [36, 37]. The screening battery includes the following measures and seven dependent variables used for exclusionary determinations: 1) total recall and 2) delayed recall from the Hopkins Verbal Learning Test—Revised ([38], 3) total score on the Block Design and 4) total score on Digit Span subtests from the Wechsler Adult Intelligence Scale-IV [39], 5) total score on the FAS Verbal Fluency Task [40], 6) total score on the Boston Naming Test [41], and 7) perseverative errors on the computerized version of the Wisconsin Card Sort Test [42]. The WAIS-IV subtests are completed between the immediate and delayed recall tasks of the Hopkins Verbal Learning Test. Descriptions of each of these measures and details on their scoring and norms are provided in Supplementary Materials. Participants will be excluded from the study due to having impairment levels consistent with dementia based on one of the following criteria: 1) if at least one normed score in both the memory and non-memory domains is < -2 *S.D.*s below the mean (i.e., T < 30), 2) if the average of their normed scores on all seven neuropsychological measures is < -2 *S.D.s* (i.e., average T < 30), or 3) if the participant reports less than intact function on all items of the Lawton Instrumental Activities of Daily Living [43] (< 8/8 points).

Participants will be classified as having MCI if they do not meet the criteria for dementia but have at least one normed score < -1.5 *S.D.*s below the mean (i.e., T < 35) or if the average of their seven normed score is < -1.5 *S.D.*s (i.e., average T < 35). Participants will be classified as cognitively normal if all of their normed scores are within 1.5 *S.D.*s of the mean and they receive a full score on the Lawton Instrumental Activities of Daily Living. These categorizations are consistent with previous clinical definitions of MCI and dementia [44, 45] and with previous research in MCI and dementia classification [36, 37]. However, an important limitation with this approach is that, although our categorization of MCI may be consistent with clinical conceptualization, it has been demonstrated that a significant minority of healthy older adults will have at least one “impaired” score < -1.5 *S.D.*s from the mean when given a neuropsychological battery [46, 47]. This has been of particular concern when trying to characterize MCI [48, 49]. In the event that the normed scores for a participant are not able to be computed due to errors in a given test’s administration, the participant will be assessed for cognitive impairment using an electronic version of the Montreal Cognitive Assessment [50] with its provided cut-off scores (26/30 or higher denoting normal cognition).

Other reasons for exclusion of potential participants include if they have been previously diagnosed with a neurological disorder, an inflammatory disorder, or a terminal illness. Participants with a history of psychotic disorders, substance abuse, or a prior learning disability will also be excluded. Additionally, participants diagnosed with a psychiatric disorder in the last two years by a mental health professional and those scoring greater than 19 on the Center for Epidemiologic Studies Depression Scale [51] will be excluded from the study. Participants currently taking medications belonging to any of the following drug classes will be excluded: sedatives, selective serotonin reuptake inhibitors, benzodiazepines, barbiturates, sedative-hypnotics, anti-inflammatories, chemotherapies, and any drugs altering brain function or enhancing cognitive performance. Anyone who has engaged in formal mindfulness training within the past year will also be ineligible or with current regular practice of meditation—defined as practicing any kind of meditation practice for greater than 10 min per day—will be ineligible. As the study involves participants engaging with practices on a web/mobile platform, participants will also be excluded from the study if they do not have access to the internet. Participants without access to computers but with internet access will be provided mini-iPads.

### Sample size

In our pilot study [34], we found a partial eta square of 0.088 for the repeated-measures ANOVA examining the effect of mindfulness on Go/No-Go performance as a function of working memory index. Based on an alpha of 0.05, a total sample size of 103 would be needed to yield an estimated power greater than 0.80. Given the attrition observed in our pilot study (~ 20%), the 12-month duration of the proposed intervention, and multiple assessment sessions, we will recruit at least 130 participants to account for a potential 25% drop-out rate. Power analysis for the imaging data was based on the parameter estimates in the Kilpatrick study [52] examining the impact of mindfulness training on functional connectivity of resting-state networks. The resulting effect sizes were *d* = 1.29 and *d* = 0.99 for auditory/salience regions To be conservative, we used the effect size of *d* = 0.99. To detect an effect of this size with 90% power, we would need 23 subjects per group for a total of 46 people. A study by Kilpatrick and colleagues [52] compared their mindfulness intervention against a wait-list control group. It is likely that the parameter estimates of their study include variance that may be accounted for by non-specific factors. In the present study, our mindfulness training group will be compared with an active control group that will control for many of the non-specific factors. Thus, assuming an effect size of *d* = 0.76, we plan on collecting MRI data on 75 participants to yield an estimated power greater than 0.90.

### Study procedures

Participants are being drawn from the greater central Ohio area. Recruitment for the study started in August 2018 and is currently ongoing. We are implementing a multi-medium recruitment strategy, advertising for the study using both printed and online communications, in-person recruitment, community outreach, and other opportunities aimed at our targeted demographic. Table 1 provides a list of our primary recruitment sources for the study. Participants interested in the study will either complete a phone screen or an online survey that is designed to collect basic demographic information, current medications, and history of physical as well as psychological health. Participants who meet basic inclusionary criteria are then invited to the Clinical Neuroscience Laboratory at The Ohio State University to complete an in-person screening session. Those meeting the study criteria as described above are invited for additional behavioral and imaging-based assessments. Table 2 lists the measures administered at pre-training, post-training, and at 6-month and 12-month follow-up sessions. Participants are also given a Qualtrics link with the following survey measures at baseline, pre-training, post-training, and the two follow-up sessions: the Mindful Attention Awareness Scale [53], the Difficulties in Emotion Regulation Scale [54], the Perceived Stress Scale [55], the World Health Organization Quality of Life – BREF [56], the Beck Anxiety Inventory [57], the Five Facet Mindfulness Questionnaire [58], the Coronavirus Anxiety Scale [59], Psychological Stress Associated with the COVID-19 Crisis [60], and the Everyday Discrimination Scale [61].

**Table 1:** List of primary recruitment sources being employed for the study

Category	Recruitment Strategy
**Printed Materials**	Flyers were displayed in recreational centers, community centers, libraries, cafes, barber shops, and churches throughout the central Ohio area
	Advertisements were displayed on Campus Area Bus Service buses at The Ohio State University
	Post cards were distributed to households in the central Ohio area
**Online Recruitment**	Research Match (an online recruitment tool with a database of participants.)
	Study Search (a database of active studies at The Ohio State University)
	Media advertisements (Facebook, Columbus Dispatch, Ohio State University’s OnCampus newspapers)
**Radio Advertisement**	Radio Stations aired an advertisement for the study (106.7 The Beat)
**Snowball Recruitment**	To facilitate oversampling of individuals from historically marginalized populations, a referral recruitment strategy was implemented. For this, enrolled participants could refer potentially eligible friends or family members to the study to receive an additional $5 per referral, up to a maximum of $35, when the individual contacted the study’s research team

**Table 2:** Measures being administered across study visits for the HealthyAgers Trial

Procedure	Visit 1 Screening	Visit 2 Pre-intervention	Visit 3 Post-intervention	Visit 4 6-month Follow-up	Visit 5 12-month Follow-up
Written Informed Consent	X				
Eligibility Criteria Assessment	X				
Cognitive Status Battery					
MCI	X				
MoCA^a^	X				
Questionnaires		X	X	X	X
Behavioral					
Go/No-Go	X^b^	X^b^	X	X	X
Conner’s CPT-3	X^b^	X^b^	X	X	X
NIHTB Cognition		X	X	X	X
ERC		X	X	X	X
NAB Driving Scenes		X	X	X	X
Neuroimaging^c^		X	X		X
Blood Draw		X	X		X

*Conner’s CPT* Conner’s Continuous Performance Test-3, *ERC* Emotion Regulation Choice task, *MCI* Mild Cognitive Impairment assessment battery, *MoCA* Montreal Cognitive Assessment, *NAB Driving Scenes* Neuropsychological Assessment Battery Driving Scenes test, *NIHTB Cognition* National Institute of Health Toolbox Cognition Battery

^a^ MoCA is only administered if an administration error occurs during the MCI assessment battery

^b^ Administration of GNG and Conner’s CPT are split between the Screening and Pre-intervention visits and are counterbalanced across participants

^c^ Participants with contraindications for the MR environment complete a non-MRI assessment

### Primary outcome

The primary outcomes for this study are changes in measures of attentional control and mind-wandering during tasks of sustained attention. To quantify both attentional control and mind-wandering the following measures are being administered:

#### Go/No-Go

In this task, participants are presented with one of two visual stimuli (“X” and “M”, or “Z” and “/”) for 750 ms followed by an inter-trial-interval of 750 ms. They are asked to press the corresponding key on frequent Go trials and to withhold responses on the less frequent (occurring 10% of the time) No-Go trials signaled by an auditory tone. Participants complete six blocks of this task with each block containing 54 Go trials, six No-Go trials, and three mind-wandering probes. The quasi-randomly presented mind-wandering probes ask participants to categorize their immediately preceding thought as: 1) “On-task”, 2) “thinking about performance on the task”, or 3) “thinking about personal worries, day-dreaming, fantasizing or just lost in thought.” The primary outcome variable for attentional control will be the signal detection sensitivity index (d_L_). The d_L_ provides a measure of task performance and will be calculated using the formula for logistic distributions: d_L_ = ln{[H(1-FA)]/[(1-H)FA]}, where H refers to hit rates for go trials and FA refers to false alarms on no-go trials. The primary outcome variable for mind-wandering will be the proportion of off-task probes endorsed by the participant. In addition, we will compute response-time variability—the trial-by-trial fluctuation in reaction time—as an indirect marker of mind-wandering.

#### Conner’s continuous performance test

In this task, participants are presented with a series of letters displayed one at a time on the screen and are instructed to press the space bar as quickly as possible for all letters (target), except for the letter ‘X’ (non-target). Participants are instructed to withhold their responses during ‘X’ letter (non-target) trials. Participants complete six blocks of 60 trials each in which letters are presented for 250 ms and inter-stimulus intervals are 1, 2, or 4 s in duration. Participants’ ability to differentiate between non-targets and targets is reflected by detectability (d’) scores and will be the primary outcome assessing attentional control. Participant d’ scores are reverse-scored such that higher *T*-scores reflect worse performance (i.e., poorer discrimination between targets and non-targets). Response time variability (RT variability), an index of mind-wandering for this task, is a measure of response speed consistency and will be calculated as the standard deviation of each participant’s response time (ms) on correct trials. Higher RT variability scores is indicative of varying attentional states and will be employed as an indirect index of mind-wandering in this task.

The efficacy of the intervention for sustained attention will be determined by improved performance on both measures. Similarly, we will conclude that mindfulness training causally impacts mind-wandering if there are reductions in both self-reported mind-wandering (i.e., on embedded probes) and in response time variability.

### Secondary outcomes

Secondary outcomes in this study include neuroimaging assessments, measures of fluid cognition, everyday cognition, and emotion regulation strategy use, and systemic markers of inflammation.

#### Neuroimaging assessment

**Fig. 2:**
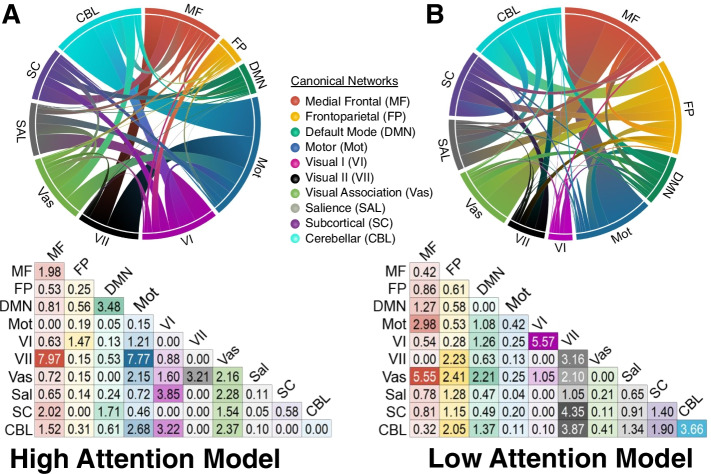
Functional neuroanatomy of the sustained attention connectome-based predictive model (saCPM). **A** Presents the involvement of the ten canonical networks in the high attention model with edges representing functional connections that positively predict attentional control. Ribbons in the ring plot visualization are proportional to the representation of each network in the saCPM model. The matrix presented on the bottom shows the relative contribution of each network to the saCPM model adjusted using the formula described in Greene et al., 2019. Panel **B** highlights the network localization of the low attentonl model with edges representing functional connections that negatively predict attentional control. The matrix on the bottom shows the relative contribution of each network in the saCPM model. Chord diagrams were generated using the software Flourish (v7.0.0, https://app.flourish.studio)

Functional MRI (fMRI) and structural MRI data will be collected at pre-training, post-training, and at 12-month follow-up. The primary dependent variable from the neuroimaging data is the network strength in a connectome-based predictive model of sustained attention (saCPM; [62]). Figure 2 presents the functional neuroanatomy of the high attention model (includes functional connections that positively predict attentional control) and the low attention model (includes functional connections that negatively predict attentional control). The saCPM model, combining the functional connections of the high and low attention models, will be employed in the current study. Network strength in this whole-brain, functional connectivity-based model of attentional control predicts performance on variegated tasks of attentional control [63–65], generalizes to predict performance across young adults [66], older adults [65], and individuals with attention-deficit hyperactivity disorder [62], and, critically, shows responsiveness to change following methylphenidate treatment for attention-deficit hyperactivity disorder [63]. Participants will perform the gradual-onset continuous performance task (gradCPT; [62, 67, 68]) while in the scanner. In this task, participants view circular images of city and mountain scenes. Participants are asked to respond to the frequently occurring city scenes (~ 90% of images) and to withhold responses to mountain scenes. Participants will complete two separate runs with each run lasting 13 min and 44 s. Neuroimaging data will be checked for quality control and preprocessed using our lab’s established pipelines [69]. The preprocessed 4D data for each participant will be parcellated into 268 contiguous and functionally defined regions using a parcellation scheme covering the cortex, subcortex, and cerebellum [70]. The connections between each pair of these nodes, defined as edges, will be computed by correlating the time-course of activity between the nodes. This will result in a 268X268 functional connectivity matrix with cells of the matrix representing the magnitude of these edges or correlations. Using the network mask of the saCPM neuromarker [63], which has been validated in our prior work with older adults [71], we will compute the network strength of the marker for each participant at pre-training, post-training, and at 12-month follow-up.

#### NIH cognitive toolbox

The cognition battery of the NIH Toolbox was designed as part of the NIH Blueprint for Neuroscience Research [72] to assess cognitive functioning across multiple domains in a standardized format. The battery is designed for use with participants between the ages of 3–85 with alternate forms available for longitudinal assessments. All tasks will be completed using fifth generation iPads with set-up and administration standardized across all participants. We will employ the fully corrected fluid cognition T-scores that norm for age, gender, education, and race/ethnicity for the following five tasks: Pattern Comparison Processing Speed, List Sort Working Memory, Picture Sequence Memory, Dimensional Change Card Sort, and Flanker Inhibitory Control and Attention. Please see Supplementary Materials for a detailed description of each of these tasks.

#### Neuropsychological Assessment Battery (NAB) driving scenes

Driving is an important functional domain that plays a critical role in the independence of older adults. To assess the impact of the intervention on functional ability, participants are being administered the Driving Scenes subtest of the NAB as a proxy measure of everyday functional ability. This measure has been shown to serve as a valid proxy for real-world driving abilities [73], it can identify at-risk drivers [74], and it is associated with global cognitive impairment in older adults with and without cognitive impairment [75]. Participants are presented with one driving scene for 30 s. This is followed by the presentation of a new scene for 60 and up to 120 s. Participants are asked to identify elements of the new scene that may be new, diferrent, or missing compared to the first scene. The main dependent variable from this task is the total number of correctly identified elements as adjusted for age, sex, and education [76].

#### Emotion regulation task

Participants complete an adapted version of the Emotion Regulation Choice Task originally developed by Sheppes and colleagues [77]. Participants are shown a series of 24 negatively valenced images and are instructed to regulate their emotions to each image using one of two emotion regulation strategies, namely acceptance or suppression. Twelve of the trials consist of low-intensity images and the remaining 12 trials consist of high-intensity images. All images were taken from the International Affective Picture System [78]. Prior to completing the task, participants undergo a brief training in which they read about each emotion regulation strategy and are asked to verbalize to the experimenter how they are using these strategies during the six practice trials. The order for which emotion regulation strategy is learned first will be counterbalanced across participants. Broadly, acceptance is defined as allowing one’s feelings to come and go without trying to control or avoid them, and suppression is defined as pushing one’s feelings down or out of one’s mind. Following the practice trials, participants proceed to the 24 task trials. Overall acceptance use will be calculated as the percentage of total trials (24 trials) in which the participant chooses to use acceptance to regulate their emotions. Acceptance use will be quantified as the percentage of low- and high-intensity images (12 trials each), respectively, in which the participant chooses to use acceptance. Scores can range between 0 and 100 percent, and higher scores reflect a greater preference for acceptance.

#### Systemic markers of inflammation

Blood samples will be collected between 8:00 am-10:00 am by a trained phlebotomist at three timepoints: pre-training, post-training, and at the 12-month follow-up session. Following standardized protocols, and implementing same control for each participant at all time points, the frozen samples for each participant will be assayed for the inflammatory markers at the same time. Various peripheral inflammatory markers exist which may be investigated for their association with cognition; however, there is strong support for C-reactive protein, interleukin-6, and tumour necrosis factor alpha to be consistently associated with cognitive outcomes in older adults [79, 80]. Additionally, these inflammatory markers appear to be reduced following mindfulness meditation interventions in a dose–response relationship [81–83]—an effect with several biologically plausible mechanisms [84–86]. This relationship appears particularly robust in older adults [87]. Although some previous studies have examined composite scores from multiple inflammatory markers, these composites are not standardized across studies and biomarkers are implicated in unique physiological processes [88–90]. Therefore, we will examine peripheral inflammation via these three markers independently.

### Randomization

Eligible and consenting participants will be randomized after completing baseline assessments to one of two groups: MBSR or LifeEd. A random permuted-block design (blocks of size 2 and 4) will be used, and the randomization with an allocation ration of 1:1 will be stratified by sex assigned at birth (male, female), cognitive status (cognitively normal, MCI), and MRI eligibility (eligible, not eligible) to ensure balance across these important factors. The biostatistician on the team, R.A., prepared and maintains the randomization sequence for the eight strata. The project coordinator has been given concealed envelopes and only after a participant completes all pre-assessment sessions and is deemed eligible for the study does the project coordinator open the envelope from the respective stratum and assign the participant to either the MBSR or the LifeEd group. The project coordinator, the study facilitators and supervisors, and the study biostatistician will be the only members of the team who will be unblinded to group assignment. All personnel conducting assessments, behavioral or neuroimaging, will remain blind to group assignment.

### Study interventions

#### Mindfulness-Based Stress Reduction (MBSR) program

The MBSR program being offered in this study is modeled after the eight-week program developed and popularized by Jon Kabat-Zinn to reduce pain and stress [24]. The MBSR program is offered in a group setting wherein participants meet with trained facilitators once a week for two and a half hours each (except for the first class which is three hours) along with a four-hour retreat offered on a weekend (between weeks six and seven)—thus resulting in 24.5 h of face-to-face training. In these classes, facilitators cover a combination of experiential practices, didactics and teachings on the topics of mindfulness, and mindful movement exercises. These practices and teachings all center on cultivating present moment awareness in a framework that promotes acceptance and non-judgment. Moreover, the participants are provided with several opportunities to engage in small and large group-based discussions that offer a platform for social support, validation, and shared experiences. Supplementary Table 1 provides the agenda for each of the eight sessions of the MBSR program as well as the practices taught during the retreat. In addition to these classes, all participants are invited to engage in 30 min of formal homework practices, delivered through our web/mobile-based, in-house application that was named after the study—HealthyAgers. Working with a team of inter-disciplinary experts, including certified mindfulness experts, psychologists, wellness coaches, and computer programmers, we have carefully designed this iOS/Android mobile/web application to deliver and track guided meditation practices. This application will allow us to collect objective practice data on all our participants with the application providing information on minutes practiced, the frequency of engagement in mindfulness practices per week, and additional meta-data, including preferred practices, time of day preference, etc. After the eight weeks of the intervention, participants will be invited to continue engagement with mindfulness practices on their own, five times per week, for 12 months with two-hour, group-based booster sessions provided by the study facilitators every three months. Prior to the pandemic, all intervention sessions were provided in our group psychotherapy rooms housed in the Department of Psychology. Sessions shifted to the Zoom platform in March 2020 and continue to be offered virtually.

#### Lifestyle Education (LifeEd) program

The LifeEd program is an active control group against which we will compare the effects of the MBSR program. Participants are provided with psychoeducation on topics related to healthy living and wellness for older adults. The series includes didactics covering the latest evidence-based science on: sedentary behavior, physical activity, cognitively stimulating activities, social support, sleep, stress management, nutrition, and hydration. This program is designed to control the many active ingredients of a group-based psychotherapeutic program, like MBSR. As such, it is matched for setting (group), number and training of facilitators, amount of time receiving in-person instruction, and amount of assigned homework time. In addition, to ensure that it is matched with respect to engagement in experiential exercises, participants are introduced to light stretching and toning exercises during the two-and-a-half-hour class sessions. These stretching and toning exercises were designed by a certified yoga instructor with expertise in working with older adults. Supplementary Table 1 also provides detailed agendas for all eight sessions of the LifeEd program. Moreover, participants in the LifeEd program also have access to the web/mobile application—HealthyAgers—with participants in this group engaging in the following combination of homework practices: light stretching and toning exercises three times/week, reading two popular press articles and answering questions related to them once a week, and watching one to two videos and answering related questions once a week. All homework materials are standardized to take ~ 30 min with all readings and videos delivered through the in-house application. Finally, like the MBSR program, participants in the LifeEd group are also being provided booster sessions once every three months with the encouragement to continue completing homework practices five times per week. Prior to the pandemic this group also met in-person with trained facilitators and transitioned to Zoom sessions starting in March 2020 because of the COVID-19 pandemic.

#### Facilitation of MBSR and LifeEd groups and treatment fidelity

Advanced graduate students in clinical psychology facilitate both the MBSR and LifeEd groups. The study PI, RSP, and her team’s mindfulness instructor, CR, provide 25 h of training for facilitating MBSR groups and another 25 h of training for facilitating LifeEd groups to advanced students with prior training in individual and group psychotherapy. Detailed training manuals, outlining the session agendas and transcripts, are provided to the students. Additionally, throughout the facilitation of these programs, new facilitators receive weekly group supervision (1.5 h.) and individual supervision (30 min). In accordance with the guidelines proposed by the Treatment Fidelity Workshop of the NIH [91] and making adaptations consistent with published guidelines for establishing fidelity for mindfulness-based interventions, [92] we will assess the fidelity for both protocols on the five identified components: design, training, delivery, receipt, and enactment. Table 3 provides details on the fidelity assessments being conducted for the current study.

**Table 3:** Fidelity components being assessed for the current study

Treatment Fidelity Component	Fidelity Assessments Being Implemented in the Current Study
**FORMAT OF DELIVERY**	
1. Rationale for MBI adaptations	1. MBSR and LifeEd materials adapted for older adults and include discussions around healthy living, barriers, challenges, and ideal outcomes
2. MBI and control group matching	2. MBSR and LifeEd sessions take place for 2.5 h (first session is for three hours), 1 × per week, for 8 weeks
3. Plan for implementation setbacks	3. Trained back-up facilitators for MBSR and control groups; scheduled website maintenance and back-up technicians for web failures
**TRAINING**	
4. Formal facilitator training	4. 25 h. of face-to-face facilitator training for MBSR and 25 h. of training control groups with MBSR instructor and PI with 22 and 12 years of experience, respectively
5. New facilitator monitoring	5. PI and MBSR consultant will provide weekly 1.5 h. group supervision + 30 min. of individual supervision
**DELIVERY**	
6. Electronic recording of program sessions	6. Research coordinator will monitor web-app delivery; all inquiry discussions will be recorded and saved
7. Program sessions reviewed and rated	7. Interventions will be delivered via the same format across the two groups; facilitators will discuss weekly group-based participant feedback at supervision
8. Facilitator supervision and feedback	8. PI and MBSR consultant will provide weekly 2-h supervision (group + individual) with new facilitators to ensure standardized delivery
**RECEIPT**	
9. Participant attendance	9. Participant attendance and meta-data will be recorded; participants who miss sessions will receive call from facilitator
**ENACTMENT**	
10. Practice adherence	10. Participants' intervention engagement and homework completion will be recorded and monitored
11. Measurement of participant skill and strategy use in real-world settings	11. Participants' engagement level will be correlated with outcome measures
12. Dispositional mindfulness measures	12. Participants will complete mindfulness scales at pre- and post-intervention (e.g., MAAS)

Treatment fidelity reporting based on NIH Behavior Change Consortium (BCC) recommendations (Bellg et al., 2004) [91] and mindfulness intervention fidelity reporting adapted from Kechter, Amaro, and Black [92]

#### Assessing and maximizing adherence

Our lab has successfully conducted two RCTs on mindfulness training, and for both those studies, we have had adherence rates of ~ 80% (Whitmoyer et al., 2020; Schirda et al., 2020). In the current study, we will implement a number of strategies that have been successful in the past to promote adherence to the intervention as well as the assessment sessions. First, as both groups in the study will engage in training that promotes and discusses healthy aging, advertising the study as a trial on mind–body interventions for healthy aging will help recruit individuals with a genuine interest in making these changes. Second, detailing the study requirements in the recruitment materials, particularly the 30 min of daily practice for eight weeks, appears to have a positive influence on adherence to homework practices and retention rates. All our advertisement materials detail this time commitment on behalf of the participants. Third, once participants are enrolled in the study, weekly attendance at the online sessions is being monitored by course facilitators and communicated to the study coordinator. For every missed session, the study coordinator connects with the participant, and either schedules them for an individual appointment with the course facilitator or sends them a summary from the missed session. For participants who wish to not continue with the intervention, every attempt will be made to complete the assessment sessions.

### Data management and analysis

All study data (with the exception of blood samples) are being stored in the PI’s laboratory located in the Psychology Building at Ohio State. The laboratory is only accessible to approved personnel. Hard data files (e.g., consent forms, neuropsychological assessment data) are stored in locked cabinets inside the laboratory, whereas electronic data files (e.g., behavioral data, self-report questionnaires, neuroimaging data) are stored on a password-protected laboratory server. De-identified participant blood samples are stored at the Clinical Research Center at The Ohio State University.

Cognitive screening data will be coded by two independent research assistants in the lab with coder disagreements resolved by an experienced research assistant. Participants’ performance scores from the Conner’s Continuous Performance Test and the National Institute of Health Toolbox Cognition battery are automatically generated from each program and will then organized on the laboratory server via manual procedures and/or custom lab scripts. Participant’s behavioral data from the Go/No-Go, Emotion Regulation Choice, Gradual-Onset Continuous Performance Task, and the N-back task will be organized and coded by two independent laboratory members using custom scripts. Neuroimaging data quality will be assessed using a validated quality control pipeline [69] prior to conducting pre-processing. Pre-processing of the neuroimaging data will be conducted by either a postdoctoral researcher or an advanced graduate student in the lab with extensive training in neuroimaging analyses. All remaining processing and coding of neuroimaging data will be completed by two independent graduate students/postdoctoral researchers. Markers of inflammation from participant’s blood samples will be processed by the Clinical Research Center at The Ohio State University.

After preprocessing of behavioral and neuroimaging data, all data will first be tested for normality, outliers, and errors. We will then compute summary statistics and compare groups’ baseline demographic and clinical characteristics; although randomization should balance the groups with respect to these characteristics, with a finite sample size, there may be some residual imbalance. Particular attention will be paid to characteristics that may be associated with outcomes (e.g., education, depression scores) as chance imbalance in these characteristics may confound the intervention effects. All statistical analyses conducted as part of the trial will be intent-to-treat. The primary outcome variables for this study are indices of attentional control and mind-wandering. To examine the immediate effects of mindfulness training, we will use a linear mixed model to account for the within-subject correlation arising from measuring each subject at two timepoints. Group (MBSR, LifeEd), Time (pre and post-training), and the Group X Time interaction will be defined as fixed effects, with covariates, when applicable (e.g., those identified as randomly differing between groups) included as fixed effects. Each participant’s intercept will be included as a random effect. To determine the maintenance effects of mindfulness meditation, we will construct a separate mixed linear model examining the long-term efficacy of mindfulness meditation with Time (pre-training, 6-month follow-up, and 12-month follow-up), Group (MBSR, LifeEd), and the Group X Time interaction effects. Secondary factors of functional connectivity, fluid and everyday cognition, emotion dysregulation and inflammation will also be examined by constructing linear mixed models.

### Adverse event reporting

Adverse events are being graded using the common grading scale proposed by the National Institutes of Health: (a) No adverse event (event is within normal limits or not clinically significant), (b) Mild adverse event that does not require treatment, (c) Moderate adverse event that was resolved with treatment, (d) Severe adverse event that resulted in an inability to carry on normal activities and required professional medical attention, (e) Life threatening or disabling adverse event, and (f) Fatal adverse event. All study personnel are trained to monitor for adverse events, and, upon notification of an adverse event, the study PI (RSP) determines the relationship of the adverse event(s) to the intervention as unrelated, possibly related, probably related, or definitely related using the standard criteria for clinical trials. For every adverse event, a written report of the adverse event is submitted to the local IRB within 10 days of the occurrence of the event. The IRB along with the PI then determines the relationship of these events to the study protocol and determines whether the adverse event was related the design of the study. If the adverse event is not related to the design of the study or is a mild adverse event related to the design of the study, these adverse events are documented along with any changes in the protocol that will be implemented to prevent future occurrences. Additionally, the event is reported to the NIH and the Data and Safety Monitor appointed for this study at the bi-annual meeting. If the adverse event is reported to be related to the study and is classified as a moderate adverse event and above, in addition to submitting a report to the IRB, a full report will be prepared and submitted within 10 days of the adverse event to the Data and Safety Monitor. This report will include a full, detailed description of the event as well as the response of the research team. If two participants experience the same adverse event, we will immediately stop recruitment for the study, prepare a report for the IRB and the Data and Safety Monitor, and incorporate any revisions to the study protocol suggested by the monitor. Additionally, annual reports will also be submitted to the IRB indicating the progress of the study, including information on enrollments, withdrawals and completions, and the occurrence of adverse events.

### Dissemination

Informed consent documents for this study include a specific statement regarding the registration of this clinical trial as well as availability of results of this clinical trial after study completion on NIH’s clinical trials platform. Per NIH Policy on the Dissemination of NIH-Funded Clinical Trial Information (NOT-OD-16–149), we have registered the proposed study on NIH’s clinical trial platform (clinicaltrials.gov # #NCT03626532) prior to enrolling the first participant. After study completion, we will also promptly report results of the clinical trial on NIH’s clinical trial platform to provide easy access to our study findings to other researchers, health care providers, and patient communities. These summary results will be submitted to NIH’s clinical trial platform no later than one year after the primary completion date. We will also be proactive, yet cautious, in our dissemination of the results of these findings through public presentations, peer-reviewed journal publications as well as communicating the results of these studies to our study participants. After publication of primary and seconday manuscripts from this dataset, all data will be de-identified, and made publicly available.

### Ethical considerations

The Ohio State Institutional Review Board has granted approval to conduct this study (IRB #2017H0223). All participants provide written informed consent and all study personnel have received training in the ethical conduct of human subject research.

## Discussion

This ongoing Phase I/II randomized controlled trial (HealthyAgers) is designed to evaluate the efficacy of mindfulness-based stress reduction, compared to a carefully designed, active control group (Lifestyle Education), in improving attentional control, reducing mind-wandering, and strengthening the functional connections subserving attentional processing in older adults. With the provision of quarterly booster sessions and the availability of a web-based and mobile application designed to promote adherence to mindfulness practices, this study will also be able to determine the longitudinal effect of engagement in mindfulness practices for maintenance of attentional benefits. Additionally, with secondary assessments involving global and everyday measures of cognition, emotion regulation, and systemic markers of inflammation, results of this study will also add to our understanding of whether mindfulness training has the potential to improve cognitive and affective health of older adults. This systematically designed trial of mindfulness meditation with its inclusion of an active control group, assessment of theoretically-driven behavioral and neural metrics of attentional control, and longitudinal follow-up will make a significant and nuanced contribution to our understanding of whether and how engagement in mindfulness meditation promotes healthy aging.

Mindfulness meditation is becoming increasingly popular because of its promise to influence several integrated metrics of overall health—attentional control [33], emotion dysregulation [93, 94], perceived and objective markers of stress [95, 96], loneliness [97, 98], systemic markers of peripheral inflammation [82], and relationship quality [99]. The adaptation of mindfulness programs to a variety of settings was also catapulted by the increasing rise of stress and mental health problems during the COVID-19 pandemic. However, many systematic and scoping reviews of the mindfulness literature have highlighted the heterogeneity of findings within contemplative sciences and have called for a more systematic and controlled study of mindfulness meditation [33, 100]. Our scoping review [33], examining the effects of mindfulness on attentional control, surveyed mindfulness training studies through the lens of five study design issues essential for establishing causality [101, 102]: 1) randomization of participants to groups; 2) inclusion of an active control group; 3) explicit attention to the reduction of demand characteristics; 4) detailed discussion of content of the intervention and control groups; and 5) implementation of study reporting guidelines (such as CONSORT). Of the 57 studies included in the review, only four met all five essential criteria—thus necessitating further and more rigorous study of mindfulness meditation for attentional control. Additionally, ten studies included an active control group and, of those, only five explicitly discussed the procedural steps and the efforts made to reduce demand characteristics in study participants.

The three most common control groups included across the studies on mindfulness meditation involved relaxation controls, education groups, and book reading groups. Importantly, factors of social support, such as collaborative discussion within group formats and engagement in cognitively stimulating activities, were highlighted as key non-specific factors influencing attention. Our control group—the Lifestyle Education Control Group—has been matched with respect to multiple metrics. Format of delivery: both protocols were delivered in-person before the COVID-19 pandemic, and we shifted to a virtual format (after the pandemic), offering classes synchronously via the Zoom platform with participants recruited in groups of six to eight for peer support. Timing: each of the eight weekly classes (plus the retreats) is matched for timing. Content: both protocols have a mix of didactics, experiential practices, social support, and community building. Peer support: qualitative analysis of focus group discussions on mindfulness-based interventions[103–105] highlight the need for participants to connect and communicate with others engaging in the interventions. Both groups have built-in dyadic and small-group discussions on topics such as weekly materials, challenges with homework practices, and overall learning in the program. Homework assignment: both programs require engagement in daily homework assignments through our in-house application for 30 min/day for five days/week.

In our study, in addition to the inclusion of this active control group, we have implemented several procedural steps to ensure that perceived expectations— participants’ beliefs regarding the efficacy of the intervention for outcome measures [33, 101]—are minimally influenced by study design, experimenter bias, and advertising materials. Inclusion of an active control group that is matched with respect to timing, duration, content, and engagement allows for the study to be advertised using common elements for the two groups rather than an explicit focus on mindfulness meditation. All our recruitment materials—print-ads, radio ads, flyers, presentations—describe the study as examining the effect of two mind–body interventions on healthy aging. Employing the definition provided by the National Center for Complementary and Integrative Health, “mind–body practices focus on the interactions among the brain, mind, body, and behavior, with the intent to use the mind to affect physical functioning and promote health,” both our recruitment materials and the content for the active control group included elements of mind–body practices. The key difference between the two groups is the active engagement with mindfulness practices as opposed to a focus on psychoeducation in the control group. In addition to reducing demand characteristics through blinded assessors, unbiased recruitment materials, and the use of an active control group can minimize their effects, it is also essential to measure these perceived expectations. In the present study, participants in both groups are completing measures of perceived expectations at the end of the first intervention session and after the respective groups have been introduced, with the eventual goal of examining differential expectations between the two groups. Finally in our attempt to examine the impact of demand characteristics on treatment gains carefully, our primary analyses will also include an association of perceived expectations with any observed gains on outcome variables.

An additional strength of this study is the inclusion of an a priori validated neuromarker of sustained attention to examine the changes in functional connectivity following training in mindfulness meditation. Although fMRI is frequently employed to examine the effects of behavioral interventions, the precise mapping of neuroimaging features relevant to target behavior is often missing. In our prior work [65], we have established the validity of this neuromarker of sustained attention—saCPM— in older adults. In this study, network strength in this established biomarker will be examined for training-related gains, with network strength in the high attention model expected to increase as a function of training and network strength in the low attention model expected to decrease. Given the centrality of attentional control to the processes of mind-wandering and emotion regulation, we hypothesize that changes in this neuromarker will mediate mindfulness-related training gains on these metrics.

In conclusion, the HealthyAgers trial will provide data on the efficacy of mindfulness meditation to improve older adults’ attentional control abilities, reduce mind-wandering, and strengthen the neural circuitry supporting attention. By virtue of enhancing the ability to sustain attention, mindfulness meditation may also improve general cognition, increase the use of acceptance-based emotion regulation strategies, and reduce overall levels of systemic inflammation. Given the aging of the baby boomer generation and the impact of reduced attentional control for activities of daily living and quality of life, training in mindfulness meditation may, in fact, be one pathway to promote healthy aging.

## Supplementary Information

**Additional file 1.**

## Data Availability

Not applicable. This is a protocol paper so there is no data employed in the manuscript.

## Abbreviations

ANOVA: Analysis of variance
Conner’s CPT: Conner’s Continuous Performance Test-3
CONSORT: Consolidated Standards of Reporting Trials
ERC: Emotion Regulation Choice task
fMRI: functional magnetic resonance imaging
grad-CPT: Gradual-onset continuous performance task
IRB: Institutional Review Board
LifeEd: Lifestyle education control group
MBSR: Mindfulness-based stress reduction
MCI: Mild cognitive impairment
MoCA: Montreal Cognitive Assessment
MRI: Magnetic resonance imaging
NAB Driving Scenes: Neuropsychological Assessment Battery Driving Scenes test
NIH: National Institutes of Health
NIHTB Cognition: National Institute of Health Toolbox Cognition Battery
PI: Principal Investigator
RCT: Randomized controlled trial
saCPM: Sustained attention connectome-based predictive model
WAIS-IV: Wechsler Adult Intelligence Scale-IV
