# Supplementary material for: Protocol for a randomized controlled trial of mindfulness-based stress reduction to improve attentional control in older adults (HealthyAgers trial)
Source: BMC Geriatr. 2022 Aug 13;22:666. doi: 10.1186/s12877-022-03334-7 (PMC9375078; doi:10.1186/s12877-022-03334-7)
Supplement: Supplementary file 1 — Additional file 1. [file 12877_2022_3334_MOESM1_ESM.zip › HealthyAgers_SupplementalInformation_04_27R1_22.docx]

Cognitive Status Screening Battery: The following tasks are being administered as part of the cognitive status screening battery.

1. Hopkins Verbal Learning Test- Revised (HVLT-R) immediate recall and delayed recall [38]: Participants are asked to memorize and recite a list of 12 nouns read orally. Four words each from one of three semantic categories (like article of clothing, gems, vegetables, buildings, etc) are read out loud for three trials. Our primary outcome measure will be the number of items correctly remembered immediately after the learning trials (immediate recall) and after approximately a 25-mins. delay period (delayed recall)
2. Block Design from the Wechsler Adult Intelligence Scale (WAIS-IV; [39]) : Block design measures perceptual reasoning and characterizes visuospatial skills. Participants are given 30-60 seconds to match the patterns on two to nine blocks to a provided picture. This task is scored first based on accuracy of the pattern matching with additional points provided for quick, accurate matches.
3. Digit Span from the Wechsler Adult Intelligence Scale (WAIS-IV; [39]): In this task, participants are presented with lists of digits, and after each presentation of the list, they are asked to repeat it as is (digit span forward), in reverse order (digit span backwards), or in numerical order (digit span sequencing). Digits are present at the rate of one per second and the “span list” increases in length as participants move through the test. The primary outcome metric for this study is the sum of correct spans totaled across all three tasks.
4. FAS Verbal Fluency Task [40]: Participants are asked to generate words beginning with the letters “F”, “A”, and “S” (one minute for each letter). Participants cannot use words beginning with the same beginning like “eat” and “eating”, and cannot use proper nouns. The primary dependent variable will be the number of correctly generated words for the three letters.
5. Boston Naming Test [41]: In this task, participants are shown line drawings of 60 common objects and are asked to label the objects. Primary measure in the BNT and this study will be the number of correctly identified pictures participants.
6. Computerized version of the Wisconsin Card Sort Test [42]: In this task, participants are asked to sort cards into different categories. Feedback is given only in terms of Yes/No answers, while the desired sort changes unannounced over time. The primary dependent variable that we will employ in the current study will be the number of preservative errors.

NIH Toolbox Cognition Battery: The following tasks are being administered as part of the NIH Toolbox Cognition Battery.

1. The Flanker Inhibitory Control and Attention Test. Participants are presented with arrows on the screen and are asked to respond to the direction of the central arrow. Congruent trials include arrows pointing in the same direction, whereas incongruent trials involve the center arrow pointing in a different direction than the flanking arrows. The task starts with four practice trials with feedback provided to the participants after each trial. This is followed by 20 test trials with the entire task lasting three minutes. The primary dependent variable from this task will be a vector combining both accuracy and reaction time data.
2. The List Sorting Working Memory Task. Participants are presented with lists of objects (1-list condition or 2-lists condition) and are asked to recall and sequence the lists in size order from smallest to largest. Each list starts with two practice trials followed by seven test trials for the 1-list condition and six test trials for the 2-list condition. The entire task lasts approximately seven minutes. The primary dependent variable employed will be the accuracy score across the two conditions.
3. The Dimensional Change Card Sort Test. This task assesses the higher-order executive functioning abilities of task switching. Participants are presented with two target pictures varying along two dimensions and are asked to sort the series of test pictures to match the target pictures. The task starts with four practice trials after which participants are presented with a mixed block of 30 test trials. Total duration for this task is four minutes. The primary variable from this task will be a vector combining both accuracy and reaction time data.
4. The Pattern Comparison Processing Speed Test. Participants are presented with two images on the screen and are asked to indicate whether the two images are same or different. The task starts with six practice trials after which participants are presented with 130 test trials and allotted a time limit of 85 seconds. Total duration for the entire task is three minutes. The primary variable will be the total number of correctly matched trials.
5. The Picture Sequence Memory Test. Participants are presented with pictured sequences of events during an activity (e.g., “Work on the farm”). They are then requested to recall the order of events in which the sequence is performed. The task starts with one practice trial followed by two test trials. This task lasts for approximately seven minutes, and the primary dependent variable is based on correctly placed adjacent pairs of events.
