# Supplementary material for: Protocol for a randomized controlled trial of mindfulness-based stress reduction to improve attentional control in older adults (HealthyAgers trial)
Source: BMC Geriatr. 2022 Aug 13;22:666. doi: 10.1186/s12877-022-03334-7 (PMC9375078; doi:10.1186/s12877-022-03334-7)
Supplement: Supplementary file 1 — Additional file 1. [file 12877_2022_3334_MOESM1_ESM.zip › Supplemental_Table 1_v2R1.docx]

Supplemental Table 1: Provides the agendas for the eight intervention sessions and the booster sessions for the MBSR and the LifeEd groups.

| **Week #** | **Mindfulness-Based Stress Reduction** | **Lifestyle Education** |
| --- | --- | --- |
| Week #1 | 1. **Settling In** (~*5 mins*)   Allow participants to arrive, settle in, and get comfortable in a seated position.   1. **Welcoming Words, and Administrative Details** (~*20 mins*)   Facilitators take attendance, introduce the study and study personnel, and discuss administrative details for how each weekly session will go.   1. **Mindfulness Awareness Experience** (~*20 mins*)   Facilitators lead participants through a meditative practice focused on cultivating the ability to be aware of one’s present moment experience.   1. **Well Meditation and Participant Introductions** (~*20 mins*)   Facilitators lead participants through a brief meditative practice that invites them to identify what brought them to the study. Participants are then invited to introduce themselves to the group and share their “why”.   1. **Defining Mindfulness and Attitudinal Foundations** (~*15 mins*)   Facilitators define mindfulness and review the history of mindfulness-based interventions. Facilitators introduces participants to the nine attitudinal foundations (non-judging, patience, beginner’s mind, trust, non-striving, acceptance, letting go, generosity and gratitude).   1. **Break** (~*15 mins*) 2. **Raisin Meditation** (~*20 mins*)   Facilitators lead participants through a mindful eating practice with a focus on employing beginner’s mind.   1. **Body Scan Practice** (~*30 mins*)   Facilitators bring participants to the floor (if able) to engage in a lying down body scan practice.   1. **Review of Homework Materials** (~*15 mins*)   Facilitators introduce participants to the HealthyAgers web-based application and walk them through the features of the application. Facilitators outline formal and informal homework assigned for Week 1.   1. **Session Ending, Group Sharing, and Expectations Questionnaire** (~2*0 mins*)   Facilitators invite participants to share one or two things they became aware of throughout the session. Facilitators review and distributes the expectations questionnaire with participants. | 1. **Settling In** (~*5 mins*)   Allow participants to arrive, settle in, and get comfortable in a seated position.   1. **Welcoming Words and Introduction** (~*10 mins*)   Facilitators take attendance, introduce the study and study personnel, and discuss administrative details for how each weekly session will go.   1. **Aims of the Study and Discussion** (~*35 mins*)   Facilitators review the aims of the study and introduce participants to the bidirectional relationships between psychological and physical health. Facilitators also review the history of psychology in medicine and the transition from the biomedical model to the biopsychosocial model.   1. **Brief Discussion of Posture, Stretching and Toning** (~*15 mins*)   Facilitators play a video introducing participants to the stretching and toning videos and outlining appropriate and safe posture for engaging in these exercises.   1. **Introduction to Blue Zones** (~*5 mins*)   Facilitators describe the Blue Zones project and connect it to the focus of the study.   1. **Break** (~*15 mins*) 2. **Blue Zones Video and Discussion** (~*55 mins*)   Facilitators play the Blue Zones video (Secrets of a Long Life) and lead participants through a discussion of what they learned.   1. **Final Stretching and Toning** (~*15 mins*)   Facilitators play the second stretching and toning video.   1. **Introduction to Next Week** (~*5 mins*)   Facilitators introduce the topic, sedentary behavior, that will be covered the following week   1. **Discussion of HealthyAgers Application and Homework Review** (~*15 mins*)   Facilitators introduce participants to the HealthyAgers web-based application and walk them through the features of the application. Facilitators outline reading/videos and stretching and toning exercises assigned for homework.   1. **Expectations Questionnaire, Session Ending** (~*5 mins*)   Facilitators explain and distribute the expectations questionnaire to participants. |
| Homework Assignments | - Body Scan (5 times) OR - Brief Body Scan (2 times a day for 5 days) | - 30-mins. of stretching and toning (3 times) - Watch the following videos and answer questions (once a week)   - Why Sitting is Bad for You   - Is Sitting the New Smoking? - Read the following article and answer questions (once a week)   - Sedentary time in older men and women: an international consensus statement and research priorities |
|  | | |
| Week # 2 | 1. **Settling In** (~*2 mins*)   Allow participants to arrive, settle in, and get comfortable in a seated position.   1. **Welcoming Awareness Practice, and Body Sweep** (~*20 mins*)   Facilitators guide participants through bringing awareness to the breath, emotions, sounds, and thoughts and then transitions into a body sweep practice.   1. **Mindful Chair Movement** (~*15 mins*)   Facilitators introduce the mindful movement practice which is incorporated throughout the 8-week program and boosters. Facilitators ensure participants have enough room/supports to engage in the practice and speak about safety and listening to one’s body. Facilitators play a recorded seated yoga practice.   1. **Homework Discussion** (~*25 mins*)   Facilitators lead participants through a paired discussion of their experiences with the body scan homework as well as the informal homework of attending to attitudinal qualities. Each paired discussion is followed by a larger group discussion where the facilitators lead inquiry.   1. **Lying Down Breath Awareness Practice** (~*20 mins*)   Facilitators lead participants through a few gentle standing stretches before bringing participants to the floor for a lying down breath awareness practice focused on exploring the breath throughout the body.   1. **Break** (~*15 mins*) 2. **Body Scan with Focus on Sensations** (~*30 mins*)   Facilitators share with participants a list of sensation words and then invites participants to return to the floor for a body scan practice focused on bringing awareness to sensations throughout the body.   1. **Sitting Meditation with Focus on Breath Awareness** (~*20 mins*)   Facilitators lead participants through final practice of the session with focus on inhabiting the breath in different areas of the body.   1. **Homework Review and Session Ending** (*~3 mins*)   Facilitators outline formal and informal homework assigned for Week 2. | 1. **Welcome and Agenda Setting** (~*5 mins*)   Allow participants to arrive, settle in, and get comfortable in a seated position. Review agenda for class with participants.   1. **Homework Review** (~*15 mins*)   Facilitators divide participants into small groups and ask participants to discuss the readings and videos they were assigned.   1. **Stretching and Toning Video** (~*30 mins*)   Facilitators play first stretching and toning video.   1. **Lecture on Sedentary Behavior** (~*60 mins*)   Facilitators present research on sedentary behavior. Specifically, facilitators discuss what sedentary behavior is, sedentary behavior patterns in older adults, the risks of engaging sedentary behavior including risks for brain health and cognition, and recommendations for engaging in less sedentary behavior. Following the lecture, facilitators lead participants through a discussion reflecting on what they learned and how it applies to their lives.   1. **Break** (~*15 mins*) 2. **Stretching and Toning Video** (~*15 mins*)   Facilitators play second stretching and toning video.   1. **Session Closing and Introduction to Next Week** (~*10 mins*)   Facilitators introduce next week’s topic (physical activity), review the assigned homework for Week 2, and answer any questions participants have. |
| Homework Assignments | - Body Scan (2 times) - Breath Awareness (3 times) | - 30-mins. of stretching and toning (3 times) - Watch the following TED talks and answer questions (once a week)   - The Disruptive Power of Exercise - Read the following articles and answer questions (once a week)   - Exercise and Physical Activity   - Physical Activity and Health   - The Brain-Body Connection |
|  |  |  |
| Week # 3 | 1. **Settling In** (~*2 mins*)   Allow participants to arrive, settle in, and get comfortable in a seated position.   1. **Welcome and Standing Movements** (~*15 mins*)   Facilitators lead participants through an opening alternate nostril breathing practice. Facilitators invite participants to come to standing and plays a standing movements video.   1. **Sitting Meditation Practice** (~*20 mins*)   Facilitators lead participants through a seated practice where participants are invited to observe the breath at the nostrils, upper chest, the belly, and the whole body.   1. **Homework Discussion** (~*25 mins*)   Facilitators lead participants through a large group discussion of their experiences with the body scan homework and a discussion of what participants became aware of when bringing attention to pleasant events.   1. **Break** (~*15 mins*) 2. **Mindful Yoga** (~*40 mins*)   Facilitators introduce and play a seated yoga video.   1. **Break** (~*5 mins*) 2. **Sitting Meditation with Focus on Sensory Awareness** (~*25 mins*)   Facilitators invite participants to take their seats and engage in a discussion on hand mudras. Facilitators introduce five different hand mudras before moving into a seated meditation focused on observing sensations.   1. **Homework Review and Session Ending** (~*3 mins*)   Facilitators outline formal and informal homework assigned for Week 3. | 1. **Welcome and Agenda Setting** (~*5 mins*)   Allow participants to arrive, settle in, and get comfortable in a seated position. Review agenda for class with participants.   1. **Homework Review** (~*15 mins*)   Facilitators divide participants into small groups and ask participants to discuss the readings and videos they were assigned.   1. **Stretching and Toning Video** (~*30 mins*)   Facilitators play first stretching and toning video.   1. **Lecture on Physical Activity** (~*60 mins*)   Facilitators present research on physical activity. Specifically, facilitators discuss what physical activity is, physical activity in older adults, the risks of not engaging in enough physical activity including risks for brain health and cognition, and recommendations for increasing physical activity. Following the lecture, facilitators lead participants through a discussion reflecting on what they learned and how it applies to their lives.   1. **Break** (~*15 mins*) 2. **Stretching and Toning Video** (~*15 mins*)   Facilitators play second stretching and toning video.   1. **Session Closing and Introduction to Next Week** (~*10 mins*)   Facilitators introduce next week’s topic (cognitively stimulating activities), review the assigned homework for Week 3, and answer any questions participants have. |
| Homework Assignments | - Breath Awareness (2 times) - Mindful Yoga (3 times) | - 30 mins of stretching and toning (3 times) - Watch the following videos and answer questions (once a week)   - Interview with Yaakov Stern   - After Watching this Your Brain Will Not be the Same - Read the following article and answer questions (once a week)   - Cognitively Stimulating Activities Readings from the Global Council on Brain Health |
|  |  |  |
| Week # 4 | 1. **Settling In** (~*2 mins*)   Allow participants to arrive, settle in, and get comfortable in a seated position.   1. **Chair Yoga and Sitting Meditation** (~*40 mins*)   Facilitators share a feelings inventory and lead a discussion on feelings before transitioning into some brief seated movements. Facilitators lead a brief body sweep and transition into a seated meditation focused on exploring sensations and emotions.   1. **Homework Discussion** (~*20 mins*)   Facilitators break participants into small groups and each facilitator leads a group through a discussion on observing unpleasant events and how the formal practices have been going. Following each small group share is a large group share covering the same topic.   1. **Introduction to Stress** (~*23 mins*)   Facilitators lead inquiry on “what stress means” and elicit responses from participants. Facilitators share didactics on types of stress (acute vs. chronic) and their physiological signatures. Facilitators allow time for sharing following the didactics and emphasize the importance of breaking the stress cycle.   1. **Break** (~*15 mins*) 2. **Mindful Movement** (~*15 mins*)   Facilitators introduce the mindful movement practice and play a video on Thich Naht Hanh movements.   1. **More on Stress** (~*30 mins*)   Facilitators introduce the repeated questioning practice and mindful listening. Facilitator pairs participants to engage in the repeated questioning exercise and models this with the other facilitator. The question is, “How do you know when you are stressed?”. Facilitators lead inquiry about the experience tying in experiences to the concept of stimulus/reaction and stimulus__response.   1. **Homework Review and Session Ending** (~*5 mins*)   Facilitators outline formal and informal homework assigned for Week 4. | 1. **Welcome and Agenda Setting** (~*5 mins*)   Allow participants to arrive, settle in, and get comfortable in a seated position. Review agenda for class with participants.   1. **Homework Review** (~*15 mins*)   Facilitators divide participants into small groups and ask participants to discuss the readings and videos they were assigned.   1. **Stretching and Toning Video** (~*30 mins*)   Facilitators play first stretching and toning video.   1. **Lecture on Cognitively Stimulating Activities** (~*60 mins*)   Facilitators present research on cognitively stimulating activities. Specifically, facilitators introduce and define the concept of brain plasticity, discuss cognition and the various types of cognitive functioning, introduce and define cognitively stimulating activities, provide examples of cognitively stimulating activities, discuss benefits of engaging in cognitively stimulating activities, and provide recommendations and practical tips for engaging in cognitively stimulating activities. Following the lecture, facilitators lead participants through a discussion reflecting on what they learned and how it applies to their lives.   1. **Break** (~*15 mins*) 2. **Stretching and Toning Video** (~*15 mins*)   Facilitators play second stretching and toning video.   1. **Session Closing and Introduction to Next Week** (~*10 mins*)   Facilitators introduce next week’s topic (social support and social engagement), review the assigned homework for Week 4, and answer any questions participants have. |
| Homework Assignments | - Mindful Yoga (2 times) - Breath Awareness (3 times) | - 30-mins. of stretching and toning (3 times) - Watch the following videos and answer questions (once a week)   - The Secret to Living Longer May be Your Social Life - Read the following article and answer questions (once a week)   - The Brain and Social Connectedness |
|  | | |
| Week #5 | 1. **Settling In** (~*2 mins*)   Allow participants to arrive, settle in, and get comfortable in a seated position.   1. **Brief Body Sweep and Sitting** **Meditation** (~*20 mins*)   Facilitators invite participants to write down a thought that has preoccupied them recently and then rip up the piece of paper to symbolize letting go. Facilitator leads participants through a brief body sweep into a seated practice focused on bringing attention to the breath.   1. **Walking Meditation** (~*25 mins*)   Facilitators introduce the walking meditation practice and invites participants to bring awareness to the sensations associated with walking. Facilitator plays walking mediation video.   1. **Homework Discussion** (~*23 mins*)   Facilitators break the group into two smaller groups, and each leads a small group discussion on the formal and informal homework before transitioning to a large group discussion.   1. **Break** (~*15 mins*) 2. **Stress and Our Contribution to It** (~*30 mins*)   Facilitators walk participants through some didactics on stress with a focus on the stress cycle. Facilitators invite participants to engage in repeated questioning in pairs around the question: “How do you contribute to your own stress?”. Facilitators invite discussion around what this practice was like and introduce two acronyms (STOP and RAIN) that encourage taking a pause when stressed.   1. **Choiceless Awareness** (~*30 mins*)   Facilitators lead participants through a choiceless awareness practice where participants are invited to open their awareness to whatever may catch their attention.   1. **Homework Review and Session Ending** (~*5 mins*)   Facilitators outline formal and informal homework assigned for Week 5. | 1. **Welcome and Agenda Setting** (~*5 mins*)   Allow participants to arrive, settle in, and get comfortable in a seated position. Review agenda for class with participants.   1. **Homework Review** (~*15 mins*)   Facilitators divide participants into small groups and ask participants to discuss the readings and videos they were assigned.   1. **Stretching and Toning Video** (~*30 mins*)   Facilitators play first stretching and toning video.   1. **Lecture on Social Engagement and Support** (~*60 mins*)   Facilitators present research on social engagement and support. Specifically, facilitators discuss what social engagement and support are, age-related changes in social engagement and support, the benefits of social engagement and support including benefits for brain health and cognition, and recommendations for increasing social engagement and support. Following the lecture, facilitators lead participants through a discussion reflecting on what they learned and how it applies to their lives.   1. **Break** (~*15 mins*) 2. **Stretching and Toning Video** (~*15 mins*)   Facilitators play second stretching and toning video.   1. **Session Closing and Introduction to Next Week** (~*10 mins*)   Facilitators introduce next week’s topic (sleep), review the assigned homework for Week 5, and answer any questions participants have. |
| Homework Assignments | - Body Scan Practice (2 times) - Choiceless Awareness (3 times) | - 30 mins of stretching and toning (3 times) - Watch the following videos and answer questions (once a week)   - Why Do We Sleep - Read the following article and answer questions (once a week)   - The Brain-Sleep Connection |
|  | | |
| Week #6 | 1. **Settling In** (~*2 mins*)   Allow participants to arrive, settle in, and get comfortable in a seated position.   1. **Qigong Practice** (~*15 mins*)   Facilitators introduce the qigong practice, an ancient practice that allows for the balancing of energies, and plays the recorded video.   1. **Sitting Meditation with Reading of the Eight Worldly Dharmas** (~*43 mins*)   Facilitators lead participants through a seated meditation with a focus on noticing the breath, sounds, thoughts, and emotions and ends with a reading of the eight worldly dharmas.   1. **Homework Review and Discussion** (~*20 mins*)   Facilitators pair participants to discuss their experiences with their use of the RAIN and STOP practices and engage in mindful listening. Facilitators bring participants back together for a large group discussion.   1. **Break** (~*15 mins*) 2. **Chair Yoga** (~*15 mins*)   Facilitators play seated yoga video.   1. **Choiceless Awareness** (~*30 mins*)   Facilitators guide participants through a five-minute silent practice followed by a discussion of five qualities nurtured while meditating (steadfastness, clear seeing, cultivation of courage, awaken to our lives, no big deal) from Pema Chodron.   1. **Homework Review & Session Ending** (~*10 mins*)   Facilitators outline formal and informal homework assigned for Week 6. Facilitators remind participants about the upcoming retreat. | 1. **Welcome and Agenda Setting** (~*5 mins*)   Allow participants to arrive, settle in, and get comfortable in a seated position. Review agenda for class with participants.   1. **Homework Review** (~*15 mins*)   Facilitators divide participants into small groups and ask participants to discuss the readings and videos they were assigned.   1. **Stretching and Toning Video** (~*30 mins*)   Facilitators play first stretching and toning video.   1. **Lecture on Sleep** (~*60 mins*)   Facilitators present research on sleep. Specifically, facilitators discuss sleep and the sleep-wake style, sleep problems and disorders, age-related changes in sleep, the benefits of sleep including benefits for brain health and cognition, and recommendations for improving sleep. Following the lecture, facilitators lead participants through a discussion reflecting on what they learned and how it applies to their lives.   1. **Break** (~*15 mins*) 2. **Stretching and Toning Video** (~*15 mins*)   Facilitators play second stretching and toning video.   1. **Session Closing and Introduction to Next Week** (~*10 mins*)   Facilitators introduce next week’s topic (diet and nutrition), review the assigned homework for Week 6, and answer any questions participants have. Facilitators remind participants about the upcoming retreat. |
| Homework Assignments | - Choiceless Awareness (5 times) | **Homework (*Week 6*):**   - 30 mins of stretching and toning (3 times) - Watch the following videos and answer questions (once a week)   - Eat for Real Change - Read the following article and answer questions (once a week)   - Brain Food |
|  | | |
| Retreat | 1. **Sitting Meditation** (~*20 mins*)   Facilitators lead participants through an opening seated meditation focused on arriving to the present moment and setting an intention for the day.   1. **Partial Sun Salutations** (~*10 mins*)   Facilitators invite participants to face the direction of the rising sun and leads participants through several rounds of partial sun salutations.   1. **Body Scan with Compassion** (~*25 mins*)   Facilitators bring participants to the floor to engage in a lying down body scan with a focus on cultivating appreciation and gratitude.   1. **Standing Movements and Walking Meditation** (*~20 mins*)   Facilitators bring participants to standing for some brief standing movements before engaging in a walking meditation.   1. **Sitting with Deliberate Breath Practice** (~*20 mins*)   Facilitators lead participants through a seated practice with a focus on directional breathing.   1. **Break** (~*15 mins*) 2. **Chair Yoga** (~*15 mins*)   Facilitators play video of seated yoga practices.   1. **Choiceless Awareness** (~*30 mins*)   Facilitators lead participants through a seated choiceless awareness practice inviting participants to attend to the breath, sensations, hearing, and thoughts before moving into an open practice and inviting participants to bring awareness to whatever arises.   1. **Lying Down Dharma Teaching** (~*15 mins*)   Facilitators bring participants to the floor to engage in a lying down practice focused on a teaching from Pema Chodron about staying with discomfort.   1. **Lunch Instructions** (~*5 mins*)   Facilitators explain the intention of lunch, which is to engage in mindful, silent eating while completing a worksheet of contemplation/reflection questions related to a challenge or difficulty they are experiencing.   1. **Lunch** (~*30 mins*)   Participants engage in mindful, silent eating.   1. **Final Sit and Close** (~*35 mins*)   Facilitators lead participants through a final seated practice focused on sustaining attention. To close, facilitators and participants read a verse about life and letting go together three times. | 1. **Welcome, Brief Introduction, and Agenda Setting** (~*5 mins*)   Facilitators welcome participants to the retreat, introduce participants to the concept of stress and why it is important, and outline the agenda for the day.   1. **Stretching & Toning** (~*20 mins*)   Facilitators play first stretching and toning video.   1. **Lecture: Defining Stress and Its Impact on Health** (~*40 mins*)   Facilitators preset research on stress. Facilitators discuss the science and physiology behind stress, ways to measure stress, how stress changes with age, why we experience stress, and the risks of excessive stress including risks for brain health and cognition.   1. **Break** (~*10 mins*) 2. **Stress: Portrait of a Killer** (~*60 mins*)   Facilitators introduce and play *Stress: Portrait of a Killer* a video from National Geographic. Following the video, facilitators break participants into small groups and lead a discussion on participants’ reactions/thoughts related to the video.   1. **Break** (~*10 mins*) 2. **Lecture: Coping with Stress** (~*20 mins*)   Facilitators present additional research on stress. Specifically, facilitators review and define coping, present different kinds of coping, and discuss interventions for stress management.   1. **Progressive Muscle Relaxation** (~*20 mins*)   Facilitators introduce a practice, progressive muscle relaxation, to help manage stress. Facilitators play recording of a progressive muscle relaxation practice. Following the practice, facilitators elicit feedback on participants’ experiences with the practice.   1. **Lunch** (~*30 mins*)   Facilitators provide participants with instructions for their social lunch. Participants are encouraged to eat lunch and socialize with each other.   1. **Lecture: Mind-Body Lifestyle Changes and Stress** (~*20 mins*)   Facilitators ask participants to reflect on ways to reduce stress in their lives. Facilitators present research on how stress relates to the other topics covered in the 8-weeks.   1. **Closing** (~*5 mins*)   Facilitators conclude the session and thank participants for their participation. |
|  | | |
| Week #7 | 1. **Settling In** (~*2 mins*)   Allow participants to arrive, settle in, and get comfortable in a seated position.   1. **Mindful Movement** (~*15 mins*)   Facilitators play Thich Nhat Hanh mindful movement video.   1. **Sitting Meditation** (~*25 mins*)   Facilitators bring participants to a seated position and lead participants through a practice focused on the 4-floor metaphor which presents mindfulness as an elevator that allows us to travel across the different floors of our felt experiences (sensation, perception, evaluation, and thinking and talking).   1. **Retreat Discussion** (~*20 mins*)   Facilitators break participants into two small groups to discuss their experience of the retreat and then brings participants back to the larger group for discussion.   1. **Partial Sun Salutations** (~*10 mins*)   Facilitators lead participants through several rounds of partial sun-salutations.   1. **Break** (~*15 mins*) 2. **Loving-Kindness Meditation** (~*60 mins*)   Facilitators lead participants through a seated practice focused on cultivating a loving kindness for oneself and others.   1. **Homework Review and Session Closing** (~*3 mins*)   Facilitators outline formal and informal homework assigned for Week 7. | 1. **Welcome and Agenda Setting** (~*5 mins*)   Allow participants to arrive, settle in, and get comfortable in a seated position. Review agenda for class with participants.   1. **Homework Review** (~*15 mins*)   Facilitators divide participants into small groups and ask participants to discuss the readings and videos they were assigned.   1. **Stretching and Toning Video** (~*30 mins*)   Facilitators play first stretching and toning video.   1. **Lecture on Diet and Nutrition** (~*60 mins*)   Facilitators present research on diet and nutrition. Specifically, facilitators define diet and nutrition and review different types of food groups and nutrients, age-related changes related to diet and nutrition, the benefits of eating a nutritious diet including benefits for brain health and cognition, and recommendations for improving diet and nutrition. Following the lecture, facilitators lead participants through a discussion reflecting on what they learned and how it applies to their lives.   1. **Break** (~*15 mins*) 2. **Stretching and Toning Video** (~*15 mins*)   Facilitators play second stretching and toning video.   1. **Session Closing and Introduction to Next Week** (~*10 mins*)   Facilitators introduce next week’s topic (hydration), review the assigned homework for Week 7, and answer any questions participants have. |
| Homework Assignments | - Any meditative practice (5 times) | - 30 mins of stretching and toning (3 times) - Watch the following videos and answer questions (once a week)   - What Would Happen if You Didn’t Drink Water   - How to Make Stress Your Friend - Read the following article and answer questions (once a week)   - Hydration in the Aging   - How Stress Affects Your Health |
|  | | |
| Week #8 | 1. **Settling In** (~*2 mins*)   Allow participants to arrive, settle in, and get comfortable in a seated position.   1. **Sitting Meditation** (~*20 mins*)   Facilitators lead participants through a seated meditation focused on settling in and bringing awareness to the present moment followed by reading a brief poem.   1. **Standing Movements and Walking Meditation** (~*10 mins*)   Facilitators lead participants through some brief standing movements before transitioning into a walking meditation.   1. **Homework Review and Discussion** (~*20 mins*)   Facilitators review homework from the previous week and break participants into dyads to engage in rerepeated questioning of the question, “How can you bring more mindfulness in your life?”. Following the dyads facilitators bring participants back to the large group for discussion.   1. **Break** (*15 mins*) 2. **Meditation Track by Jon Kabat-Zinn** (~*33 mins*)   Facilitators play audio recording of Jon Kabat-Zinn discussing ways to keep mindfulness alive in one’s day-to-day life.   1. **Mountain Meditation and Reflection** (~*40 mins*)   Facilitators lead participants through a seated practice focused on embodying the deep-rootedness and stability of a mountain. Following the practice, facilitators lead inquiry focused on participants experience of the mountain meditation and how they can keep mindfulness alive in their lives’.   1. **Closing** (~*10 mins*)   Facilitators lead participants through a brief final seated practice focused on being here, open, and awake. | 1. **Welcome and Agenda Setting** (~*5 mins*)   Allow participants to arrive, settle in, and get comfortable in a seated position. Review agenda for class with participants.   1. **Homework Review** (~*15 mins*)   Facilitators ask participants to reflect on their homework and lead participants through a large group discussion.   1. **Stretching and Toning Video** (~*30 mins*)   Facilitators play first stretching and toning video.   1. **Lecture on Hydration** (~2*5 mins*)   Facilitators present research on hydration. Specifically, facilitators discuss hydration, dehydration and over-hydration, functions of water, age-related changes in hydration, the benefits of hydration including benefits for brain health and cognition, and recommendations for increasing hydration. Following the lecture, facilitators lead participants through a discussion reflecting on what they learned and how it applies to their lives.   1. **Break** (~*15 mins*) 2. **8-Week Program Review** (~*30 mins*)   Facilitators review the topics covered during the 8-week intervention and review the bidirectional relationship between psychological and physical health.   1. **Stretching and Toning Video** (~*15 mins*)   Facilitators play second stretching and toning video.   1. **Session Closing** (~15 *mins*)   Facilitators thank participants for their participation so far and invite them to return for the booster sessions. |
| Homework Assignments | - Any meditative practice (5 times) | - 30 mins of stretching and toning (5 times) |
| **Booster #** | **Mindfulness-Based Stress Reduction** | **Lifestyle Education** |
| Booster # 1 | 1. **Welcoming Words** (~*10 mins*)   Facilitators welcome participants to the first booster session. Facilitators review the intentions for the booster sessions (to ground in the meditation practices, offer space for discussions, and expand toolbox of practices).   1. **Mindfulness Movement** (~*10 mins*)   Facilitators lead participants through some brief standing movements.   1. **Spacious Breath Awareness Practice** (~20 *mins*)   Facilitators lead participants through a meditation focused on cultivating a spacious silence for the breath.   1. **Reflections on Continued Engagement with the Practices** (~*35 mins*)   Facilitators break participants into small groups to discuss participants’ experiences with the formal meditations and the informal practices. Following each smaller group discussion, facilitators bring participants back together for a larger group discussion.   1. **Thich Nhat Hanh Movements** (~*5 mins*)   Facilitators bring participants to standing to engage in a few minutes of Thich Nhat Hanh movements.   1. **RAIN of Self Compassion** (~*25 mins*)   Facilitators lead participants through a meditation focused on using RAIN to cultivate self-compassion. Following the practice, facilitators elicit feedback on this experience from the participants.   1. **Closing and Next Steps** (~*15 mins*)   Facilitators bring participant together for a final brief seated practice focused on bringing peace to oneself and others. | 1. **Welcome and Agenda Setting** (~*5 mins*)   Facilitators welcome participants to the first booster session. Facilitators review the agenda for the session and invite participants to share one thing they did for their physical or mental health recently.   1. **Booster Period Review** (~*15 mins*)   Facilitators review the intention of the booster sessions and outline the topics that will be covered. Facilitators break participants into small groups to discuss how they have been incorporating physical activity and limiting sedentary behavior into their lives over the past three months.   1. **Stretching and Toning Video** (~*30 mins*)   Facilitators play first stretching and toning video.   1. **Review of 8-Week Program** (~*5 mins*)   Facilitators review the topics covered in the 8-week intervention and the bidirectional relationship between psychological and physical health.   1. **Lecture on Sedentary Behavior** (~*20 mins*)   Facilitators review the research covered on sedentary behavior and introduce new material on sedentary behavior.   1. **Break** (~*5 mins*) 2. **Lecture on Physical Activity** (~*20 mins*)   Facilitators review the research covered on physical activity and introduce new material on physical activity.   1. **The Next Three Months** (~*5 mins*)   Facilitators inform participants of what topics (stress management and social support) will be covered at the next booster session/what they should focus on for the next three months.   1. **Stretching and Toning Video** (~*15 mins*)   Facilitators play second stretching and toning video. |
|  | | |
| Booster # 2 | 1. **Welcoming Words** (~*10 mins*)   Facilitators welcome participants to the second booster session. Facilitators ask participants to take their seats and invite them to bring awareness to the current state of their being.   1. **Mindfulness Movement** (~*10 mins*)   Facilitators bring participants to standing to engage in some brief mindful movements.   1. **Spacious Breath Awareness Practice** (~*15 mins*)   Facilitators lead participants through a spacious breath awareness practice designed to provide participants with a mostly silent space to explore the breath.   1. **Reflections on Continued Engagement with the Practices and the “Living It”** (~*35 mins*)   Facilitators break participants into small groups to discuss participants’ experiences with the formal meditations and the informal practices. Following each smaller group discussion, facilitators bring participants back together for a larger group discussion.   1. **Walking Meditation** (~*10 mins*)   Facilitators bring participants to standing to engage in a brief walking meditation focused on bringing awareness to the sensations of walking.   1. **Living Gratitude – In and Out** (~*25 mins*)   Facilitators lead participants through a seated practice focused on cultivating gratitude and giving thanks. Following the practice, facilitators elicit feedback on this experience from the participants.   1. **Closing and Next Steps** (~*15 mins*)   Facilitators lead participants through a final seated practice focused on sitting with gratitude and setting an intention for the upcoming months. | 1. **Welcome and Agenda Setting** (~*5 mins*)   Facilitators welcome participants to the second booster session and review the agenda.   1. **Booster Period Review** (~*15 mins*)   Facilitators review the intention of the booster sessions and outline the topics that will be covered. Facilitators break participants into small groups to discuss how they have been incorporating stress management and social support into their lives over the past three months.   1. **Stretching and Toning Video** (~*30 mins*)   Facilitators play first stretching and toning video.   1. **Review of 8-Week Program** (~*5 mins*)   Facilitators review the topics covered in the 8-week intervention and the study timeline.   1. **Lecture on Stress** (~*20 mins*)   Facilitators review the research covered on stress and introduce new material on stress.   1. **Break** (~*5 mins*) 2. **Lecture on Social Support** (~*20 mins*)   Facilitators review the research covered on social support and introduce new material on social support.   1. **The Next Three Months** (~*5 mins*)   Facilitators inform participants of what topics (sleep and cognitively stimulating activities) will be covered at the next booster session/what they should focus on for the next three months.   1. **Stretching and Toning Video** (~*15 mins*)   Facilitators play second stretching and toning video. |
|  | | |
| Booster # 3 | 1. **Welcoming Words** (~*10 mins*)   Facilitators welcome participants to the third booster session.   1. **Mindfulness Movement** (~*15 mins*)   Facilitators bring participants to standing and lead them through some brief standing movements.   1. **Breath, Sound, Body, and Gratitude Meditation** (~*15 mins*)   Facilitators lead participants through a seated practice focused on establishing the breath as an anchor and then expanding awareness to sound and the body before sitting with gratitude.   1. **Reflections on Continued Engagement with the Practices and the “Living It”** (~*30 mins*)   Facilitators break participants into small groups to discuss participants’ experiences with the formal meditations and the informal practices. Following each smaller group discussion, facilitators bring participants back together for a larger group discussion.   1. **Standing Yoga** (~10 *mins*)   Facilitators bring participants to standing for some brief standing yoga movements.   1. **Tonglen** (~*35 mins*)   Facilitators lead participants through a tonglen practice which is a meditative practice centered around giving and taking or cultivating compassion. Following the practice, facilitators elicit feedback on this experience from the participants.   1. **Closing and Next Steps** (~*5 mins*)   Facilitators lead participants through a final brief seated practice focused on loving kindness. | 1. **Welcome and Agenda Setting** (~*5 mins*)   Facilitators welcome participants to the third booster session and review the agenda.   1. **Booster Period Review** (~*15 mins*)   Facilitators review the intention of the booster sessions and outline the topics that will be covered. Facilitators break participants into small groups to discuss how they have been incorporating sleep hygiene and cognitively stimulating activities into their lives over the past three months.   1. **Stretching and Toning Video** (~*30 mins*)   Facilitators play first stretching and toning video.   1. **Review of 8-Week Program** (~*5 mins*)   Facilitators review the topics covered in the 8-week intervention and the study timeline.   1. **Lecture on Sleep** (~*20 mins*)   Facilitators review the research covered on sleep and introduce new material on sleep.   1. **Break** (~*5 mins*) 2. **Lecture on Cognitively Stimulating Activities** (~*20 mins*)   Facilitators review the research covered on cognitively stimulating activities and introduce new material on cognitively stimulating activities.   1. **The Next Three Months** (~*5 mins*)   Facilitators inform participants of what topics (nutrition and hydration) will be covered at the next booster session/what they should focus on for the next three months.   1. **Stretching and Toning Video** (~*15 mins*)   Facilitators play second stretching and toning video. |
|  | | |
| Booster # 4 | 1. **Welcoming Words** (~*20 mins*)   Facilitator welcomes participants to final booster session. Facilitators invite participants to arrive to the present moment.   1. **Sitting Meditation** (~*15 mins*)   Facilitator leads participants through a choiceless awareness practice with long periods of silence and limited guidance.   1. **Reflections on Continued Engagement with the Practices and the “Living It”** (~*30 mins*)   Facilitators break participants into small groups to discuss participants’ experiences with the formal meditations and the informal practices. Following each smaller group discussion, facilitators bring participants back together for a larger group discussion.   1. **Standing Yoga** (~*5 mins*)   Facilitator leads participants through some brief standing movements.   1. **Everyday Life is the Practice** (~*40 mins*)   Facilitator leads participants through a seated practice focused on cultivating the attitudinal qualities into one’s mindfulness practice and everyday life.   1. **Closing and Next Steps** (~*10 mins*)   Facilitators invite participants to sit for a final few minute of silence. To close, facilitators and participants read together a few statements on offering loving kindness to oneself and others. | 1. **Welcome and Agenda Setting** (~*5 mins*)   Facilitators welcome participants to the final booster session and review the agenda.   1. **Booster Period Review** (~*15 mins*)   Facilitators review the intention of the booster sessions and outline the topics that will be covered. Facilitators break participants into small groups to discuss how they have been incorporating nutrition and hydration into their lives over the past three months.   1. **Stretching and Toning Video** (~*30 mins*)   Facilitators play first stretching and toning video.   1. **Review of 8-Week Program** (~*5 mins*)   Facilitators review the topics covered in the 8-week intervention and the study timeline.   1. **Lecture on Nutrition** (~*20 mins*)   Facilitators review the research covered on nutrition and introduce new material on nutrition.   1. **Break** (~*5 mins*) 2. **Lecture on Hydration** (~*20 mins*)   Facilitators review the research covered on hydration and introduce new material on hydration.   1. **The End of the Study** (~*5 mins*)   Facilitators show participants how to access practices from the toolbox in the mobile application moving forward after the study is complete.   1. **Stretching and Toning Video** (~*15 mins*)   Facilitators play second stretching and toning video. |
